# Effects of emotion on prospection during decision-making

**DOI:** 10.3389/fpsyg.2014.00591

**Published:** 2014-06-23

**Authors:** Darrell A. Worthy, Kaileigh A. Byrne, Sherecce Fields

**Affiliations:** Department of Psychology, Texas A&M UniversityCollege Station, TX, USA

**Keywords:** emotions, worry, decision making, prospection, delay discounting

## Abstract

In two experiments we examined the role of emotion, specifically worry, anxiety, and mood, on prospection during decision-making. Worry is a particularly relevant emotion to study in the context of prospection because high levels of worry may make individuals more aversive toward the uncertainty associated with the prospect of obtaining future improvements in rewards or states. Thus, high levels of worry might lead to reduced prospection during decision-making and enhance preference for immediate over delayed rewards. In Experiment 1 participants performed a two-choice dynamic decision-making task where they were required to choose between one option (the decreasing option) which provided larger immediate rewards but declines in future states, and another option (the increasing option) which provided smaller immediate rewards but improvements in future states, making it the optimal choice. High levels of worry were associated with poorer performance in the task. Additionally, fits of a sophisticated reinforcement-learning model that incorporated both reward-based and state-based information suggested that individuals reporting high levels of worry gave greater weight to the immediate rewards they would receive on each trial than to the degree to which each action would lead to improvements in their future state. In Experiment 2 we found that high levels of worry were associated with greater delay discounting using a standard delay discounting task. Combined, the results suggest that high levels of worry are associated with reduced prospection during decision-making. We attribute these results to high worriers' aversion toward the greater uncertainty associated with attempting to improve future rewards than to maximize immediate reward. These results have implications for researchers interested in the effects of emotion on cognition, and suggest that emotion strongly affects the focus on temporal outcomes during decision-making.

## Effects of emotion on prospection during decision-making

Decision-making is an important task that people must engage in on a daily basis, and individuals vary in the degree of negative emotions like worry and anxiety that they experience in decision-making situations. Decisions can have both immediate and long-term effects. An individual is often faced with a choice between one option that seems more appealing at the moment and another option that, while not as immediately rewarding, is the better choice in the long-run. For example, a college graduate must decide whether to seek an immediate job that offers a decent salary or to attend some type of graduate school that will likely lead to a smaller income over the next few years, but a larger income over the course of one's lifetime. Similarly, one could spend one's annual bonus on purchasing something now or invest the money and have more to spend later. Thus, the tradeoff between maximizing immediate reward vs. seeking greater delayed reward is pervasive to many decision-making situations. Many of these situations require prospection or the representation and consideration of the future value of decisions (Peters and Buchel, 2011; Pezzulo and Rigoli, 2011; Gerlach et al., 2014). One important issue that we address in the present work is how specific emotions like worry, anxiety, and mood affect prospection during decision-making.

Emotions can become intertwined in the decision-making process. For example, both immediate and expected emotions can influence the way in which a person makes his or her decisions. In response to a new or threatening situation, a person may have an immediate emotional reaction that is experienced at the time of the decision (Loewenstein and Lerner, 2003). For example, if a person goes into the doctor for a routine check-up and is told that they need to have immediate surgery, an immediate negative response, such as anxiety, may be evoked and influence their behavior. On the other hand, a person may be told he or she needs a medical procedure in the future, and anticipatory emotions, such as worry, may be evoked. A person may have expected consequences of the event that influence their decision and future emotional reactions (Loewenstein and Lerner, 2003). Similarly, prospection is a phenomenon in which a person simulates the positive and negative consequences of a future event. A person may pre-experience the event and pre-feel anticipated emotions for the event. These simulations are influenced by previous experience and memories of similar events and the context in which they occur (Gilbert and Wilson, 2007). Thus, emotions are essential components to the prospection simulation process and understanding how specific emotions predict prospection responses is important in decision-making.

In examining the effect of emotions on prospection, it should be noted that anxiety and worry are positively correlated, but distinct affective constructs. Anxiety is characterized by somatic feelings of panic and distress, rumination, and a mental and physical over-reaction to a perceived stressor (e.g., Cook et al., 1988; Reiss, 1991; Schmidt et al., 1997; Erskine et al., 2007). Worry can be defined as a mental preoccupation with potential negative events that may occur in the future (Mueller et al., 2010). Furthermore, worry is characterized as future-oriented in that individuals tend to worry about events that could occur in the future (Borkovec et al., 1983; Brown et al., 1993). The role of worry in decision-making situations that require prospection is an important issue to address as high worriers could be predicted to be either more or less future-oriented during decision-making. One possibility is that high levels of worry are associated with more future-oriented decision-making, and worry enhances prospection during decision-making (Borkovec et al., 1983; Brown et al., 1993). Thus, high levels of worry may be associated with enhanced preference for the future, rather than the immediate consequences of each action due to a more future-oriented focus in high-worry individuals. Another possibility, which we outline below, is that worry is associated with greater intolerance of uncertainty and high-worry individuals might prefer to maximize immediate reward because there is greater uncertainty surrounding the possibility of improving future rewards or states. By examining anxiety and mood, a state rather than trait affective emotion, in addition to worry we seek to determine whether these emotions have distinct effects on prospection during decision-making or whether negative affect in general influences one's decisions.

The decision-making literature distinguishes decision-making situations involving risk from those involving uncertainty (Johnson and Busemeyer, 2010). In situations involving risk outcomes associated with each action are known along with the probabilities of obtaining each outcome. For example option A may offer a 100% percent chance of gaining $10, while option B may offer a 50% chance of gaining $50 but also confers a 50% chance of losing $30. Thus, the probabilities associated with each outcome are known, but selecting option B would confer the risk of a loss. In situations involving uncertainty the outcomes and probability distributions associated with each action are unknown and must be learned from experience. One important point to note is that decision-making situations can contain different degrees of both risk and uncertainty. Risk is usually characterized by known probabilities and rewards associated with each action, with options conferring smaller chances of gains and/or greater chances of losses deemed as riskier, while uncertainty is usually characterized by unknown probabilities and rewards associated with each action, with options with less well-known outcomes being deemed more uncertain.

Anxiety has been associated with reduced tolerance for risk during decision-making situations (Raghunathan and Pham, 1999; Maner and Schmidt, 2006). Negative emotions like anxiety have been associated with an increased preference for low-risk options during decision-making. For example, in the Balloon Analog Risk Task (BART) participants earn more money the more they pump up a balloon, but they also risk having the balloon pop if too many pumps are made (Lejuez et al., 2002). Highly anxious participants tend to pump the balloon a fewer number of times than less anxious individuals (Maner et al., 2007). Thus, in the course of the task highly anxious individuals tend to favor the less risky alternative of stopping sooner than the more risky alternative of trying another pump. Anxious decision-makers are also more likely to choose options that confer high-probability small rewards over low-probability large rewards than less anxious individuals (Raghunathan and Pham, 1999).

While anxiety has been associated with risk avoidant behavior, worry has been associated with an increased intolerance of uncertainty (Ladoucer et al., 1997; Dugas et al., 1998; Buhr and Dugas, 2006). This link between intolerance of uncertainty and worry has remained strong even when accounting for other factors often associated with worry like anxiety and depression (Freeston et al., 1994; Dugas et al., 1997; Buhr and Dugas, 2002). Temporal delays in which decision outcomes will be known can create uncertainty (Luhmann et al., 2008). Decision makers may find temporally extended periods of uncertainty as to what the outcomes of each decision are to be averse, and the tendency to avoid long periods of uncertainty may be more pronounced in participants with high levels of worry (Luhmann et al., 2008). Recently, Luhmann and colleagues examined temporal uncertainty by having participants choose from an option that offered a 50% chance of immediate reward or an option that would provide a 70% chance of reward after a delay of 5–25 s. Participants who reported higher intolerance of uncertainty were more likely to select the immediately rewarding option than participants with lower intolerance of uncertainty. Thus, intolerance of uncertainty was associated with an enhanced preference for an option that provided an immediate outcome (50%) even though this option was riskier in that it provided a lower probability of reward than the delayed outcome option (70%). In this case, intolerance of uncertainty was related to uncertainty about *when* the outcome would be known rather than *what* the outcome would be.

Uncertainty as to when decision outcomes will be known may be very relevant to the concept of prospection, as prospection involves thinking about how actions may or may not lead to improvements in one's future state. Options that lead to better immediate outcomes may be viewed with greater certainty than options that offer the prospect of improving one's future state. The possibility of future rewards from each action likely seems less certain than the possibility of immediate rewards. Maximizing immediate reward or “taking more now” involves less uncertainty than selecting an option that offers the prospect of obtaining a larger reward at a later point in time. Given the increased intolerance of uncertainty for high worriers, we predicted that high levels of worry would reduce prospection regarding how each action would lead to future reward and that high worriers would show enhanced preferences for maximizing immediate reward and reduced preferences for seeking larger delayed rewards, relative to low worriers. In the current work, we seek to directly examine this hypothesis in two different decision-making tasks that both involve prospection.

**Figure 1:**
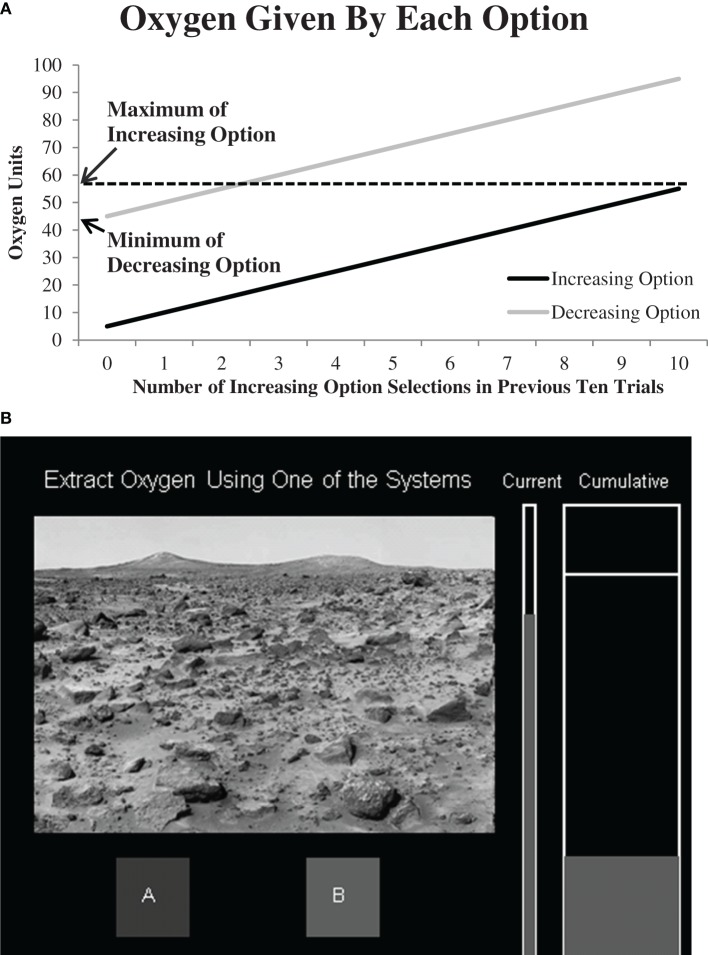
**(A)** Oxygen given by each option. The amount of oxygen given by each system was a direct function of the number of times the increasing option had been selected over the previous ten trials. “Maximum of increasing option” indicates how much oxygen was given if the increasing option had been selected on all ten of the previous trials, and “Minimum of decreasing option” indicates how much oxygen was given if the decreasing option had been selected on all ten of the previous trials. **(B)** Sample screen shot from the experiment. Participants were given a cover story where they were asked to test two oxygen-extraction systems on the Martian landscape. The oxygen extracted on each trial was shown in the “Current” tank and then transferred to the “Cumulative” tank.

In Experiment 1 we examine how worry, anxiety and mood affect behavior in the “Mars Farming Task” where good performance requires considering how each action affects both immediate and delayed rewards given by each option (Gureckis and Love, 2009; Worthy et al., 2011, 2012). This task involves decision-making under uncertainty where participants must repeatedly select from the available options to learn which option leads to the best outcome. In the task, participants are given a hypothetical scenario where they are astronauts testing two oxygen extraction systems that farm oxygen from the Martian atmosphere. The amount of oxygen extracted on a given trial is shown in Figure 1A. The amount given by each system is dependent on the previous actions the participant has made. The “increasing” option always gives a smaller amount of oxygen on each trial, but it is the optimal choice because selecting it causes rewards for both options to increase on future trials. Thus, selecting the increasing option will improve individual's future state. The “decreasing” option always gives a larger amount of oxygen on each trial, but selecting it causes future rewards to decrease, making it the sub-optimal choice, or leads to declines in individuals' future state. Thus, the option that provides the largest immediate reward (the decreasing option) must be avoided in favor of an option that provides larger rewards on future trials the more often it is selected (the increasing option). We predicted that individuals that report high levels of worry would be more likely than low-worry individuals to repeatedly select the decreasing option because it would be viewed with less uncertainty than the increasing option.

The task we use in Experiment 1 also allows us to apply a sophisticated reinforcement learning model that assumes that participants make decisions by weighing the degree to which selecting each option will maximize immediate reward or improve the participant's future state. Applying this model to the data allows us to directly infer the degree to which participants engaged in prospection-based processing whether they valued each option based on its ability to improve their future state.

In Experiment 2 we examine the role of negative emotions in a Delay Discounting Questionnaire task (DDQ; Richards et al., 1999). This task serves as a conceptual replication of the Mars Farming Task used in Experiment 1, and directly addresses whether worry, anxiety, and mood affect preferences for immediate vs. delayed reward. In the DDQ used in Experiment 2, participants are directly queried regarding their preferences for smaller amounts of money that would be given immediately vs. larger amounts of money that would be given after a delayed period of time (e.g., “would you prefer $5 now or $10 in 30 days”). Greater preference for immediate reward is indicative of greater *discounting* of delayed reward. We predicted that high worriers would view delayed rewards with greater uncertainty, and, as a result, would discount delayed rewards more than low worriers.

The two tasks we use across both experiments complement each other well. The dynamic decision-making task involves decision-making from experience, where nothing is initially known about the rewards provided by each option and participants must learn from experience. In contrast, the DDQ used in Experiment 2 involves decision-making from description where the rewards given by each option are made clear, and participants have to indicate which option they prefer (Hertwig et al., 2004). This is an important distinction because Experiment 1 requires learning about the rewards associated with each action, while in Experiment 2 participants have full information about the rewards given by each option and simply have to state their preference. However, both experiments address the juxtaposition between immediate and delayed reward which is relevant to many decision-making situations.

Enhanced prospection should be evidenced by greater preference for the increasing option in Experiment 1, and by reduced discounting of delayed rewards in Experiment 2. Additionally, enhanced prospection should be indicated by model-fitting results that suggest greater attention to improving one's future state over maximizing immediate reward. All of these measures should indicate a more “forward-thinking” approach to the decision-making tasks which is consistent with the idea of prospection. We predicted these measures that are indicative of enhanced prospection would be reduced for high worriers across both experiments.

## Experiment 1

### Methods

#### Participants

Fifty-six young adults enrolled at Texas A&M University completed the experiment for partial fulfillment of a course requirement. Participants were divided into high and low-worry groups based on a median split of Penn State Worry Questionnaire (PSWQ) scores (detailed below).

#### Materials and procedure

Participants performed the Experiment on PCs using Psychtoolbox for Matlab (version 2.5). The experiment was approved by the Institutional Review Board. Participants were asked to complete a number of questionnaires before the experiment began. Participants first completed the PSWQ (Meyer et al., 1990). The PSWQ is a 16-item scale that has demonstrated high internal consistency and test-retest reliability and has been used in several studies to measure self-reported worry (Meyer et al., 1990; van Rijsoort et al., 1999).

Participants also completed the Positive and Negative Affect Schedule (PANAS; Watson et al., 1988) and the Beck Anxiety Inventory (BAI; Beck et al., 1988) to examine the roles of affect and anxiety, in addition to worry. The BAI is a 21-item scale that has shown high internal consistency and test-retest reliability and some studies suggest that is has better convergent and discriminant validity than other anxiety scales like the State-Trait Anxiety Inventory (Frydrich, 1992; Kabacoff et al., 1997). The PANAS consists of two 10 items scales that separately measure positive and negative affect. The two scales have high internal consistency, are not highly correlated, and also demonstrate good test-retest reliability (Watson et al., 1988).

After completing the questionnaires participants were then given instructions for the decision-making task. Figure 1B shows a sample screen-shot from the experiment. Participants were given a cover story that they would be testing two extraction systems that farmed oxygen on Mars, and their goal was to extract as much oxygen as possible. A similar paradigm has been used elsewhere to examine other issues in decision-making (Gureckis and Love, 2009; Otto et al., 2009; Worthy et al., 2011, 2012). On each trial, participants were told to “collect oxygen using one of the two systems” which appeared at the top of the screen. They were allowed as much time as they wished to make a response. After they had selected one of the systems, the amount of oxygen they received for that trial was indicated in the narrow tank labeled “Current,” and after another 1000 ms the oxygen would be emptied into the “Cumulative” tank. After 2000 ms the next trial would begin.

Participants performed a total of 250 trials in the task. The rewards they received were based on the reward structure shown in Figure 1A. Rewards were a function of the number of times participants had selected the increasing option over the previous ten trials. Thus, there was a “moving window” which kept a count of the number of times the increasing option was selected over the previous ten trials. All participants began the experiment at the mid-point (3) on the x-axis.

A line on the larger tank corresponded to the amount of oxygen needed to sustain life on Mars. Participants were given the goal of trying to collect this amount of oxygen over the course of the experiment. The goal line was set at the equivalent of 18,000 points. This corresponded to selecting the optimal, increasing option on roughly 80% of trials. Participants were told nothing about the rewards available for each option or how the rewards given were dependent on how often they had selected the increasing option, but had to learn what rewards were given by each option from experience.

### Results

#### Behavioral analyses

The total oxygen collected in the task and the proportion of trials participants selected the increasing option were computed. There was a nearly perfect correlation between these two measures (*r* = 0.99, *p* < 0.001). This was expected as the amount of oxygen collected was directly dependent on how often participants selected the increasing option. Because of this association, the proportion of trials that participants selected the increasing option was used as the primary dependent variable that indicated good performance in the task.

**Figure 2:**
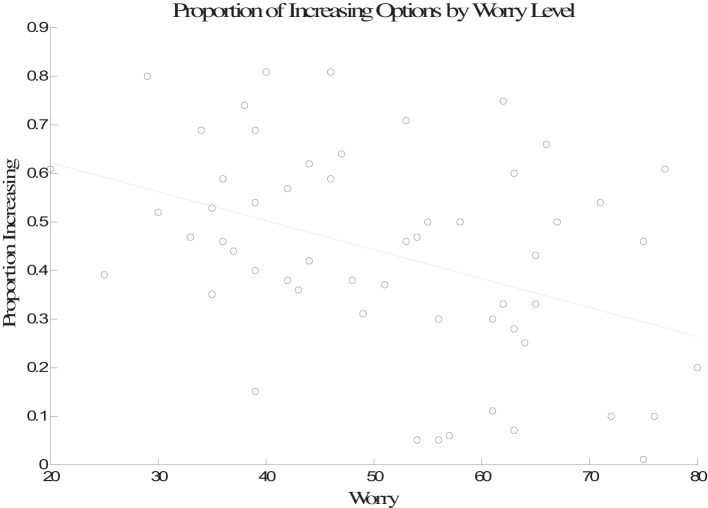
**Performance (proportion in increasing option selections) plotted as a function of worry**.

Next, the worry, anxiety, and positive and negative affect scores for each participant were entered as predictors in a linear regression with proportion of increasing option selections used as the outcome variable. Table 1 shows the correlations between the proportion of increasing option selections and each individual difference measure. There was no relationship between anxiety, negative mood, or positive mood and proportion of increasing option selections. However, there was a significant negative association between worry and proportion of increasing option selections. Additionally worry, anxiety, and negative mood were all positively correlated with one another. The results of the multiple regression indicate that worry was the only variable that was a significant predictor of performance in the task (β = −0.45, *p* < 0.01; negative mood, β = 0.00, *p* > 0.10; positive mood, β = −0.05, *p* > 0.10; anxiety, β = 0.06, *p* > 0.10). The partial correlation between worry and performance was also significant (*r* = −0.37, *p* < 0.001). The association between worry and performance is plotted in Figure 2.

**Table 1:** **Correlations Between Performance and Individual Differences in Experiment 1**.

		**1**	**2**	**3**	**4**	**5**
1	Performance	–	–	–	–	–
2	Worry	−0.41^**^	–	–	–	–
3	Anxiety	−0.12	0.39^**^	–	–	–
4	Negative mood	−0.15	0.34^**^	0.28^*^	–	–
5	Positive mood	0.09	−0.34^**^	−0.07	0.13	–

*Performance was measured as the proportion of times participants selected the increasing option throughout the task*.

* *p < 0.05*,

** *p < 0.01*.

Having established that PSWQ scores were negatively associated with performance and that these scores were the most significant predictor of performance, participants were split into high and low-worry groups based on a median split (Median = 50; Range = 20–80). Twenty-eight participants were placed in each of the low-worry and high-worry groups.

**Figure 3:**
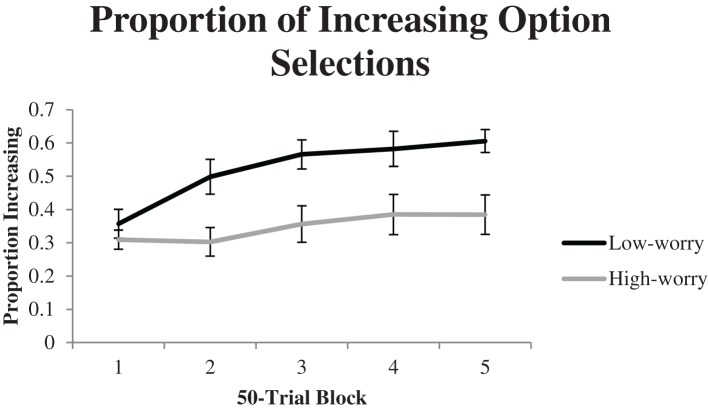
**Average proportion of increasing option selection for each group during each 50-trial block of Experiment 1**. Error bars represent standard errors of the mean.

The proportion of times participants selected the increasing option was examined in 50-trial blocks of the task. These are plotted in Figure 3. A 2 (group) × 5 (block) repeated measures ANOVA was conducted and linear effects of block were examined. There was a significant linear trend for block, *F*_(1, 54)_ = 16.40, *p* < 0.001, partial η^2^ = 0.23, and there was a marginally significant block × group interaction, *F*_(1, 54)_ = 3.02, *p* < 0.10, partial η^2^ = 0.05. There was also a significant effect of group, *F*_(1, 54)_ = 10.95, *p* < 0.01, partial η^2^ = 0.17. Over all trials participants in the low-worry group (*M* = 0.52, *SE* = 0.04) selected the increasing option more often than participants in the high-worry group (*M* = 0.34, *SE* = 0.05). To analyze the locus of the block × group interaction, we examined the linear trend for block within each group. The linear trend for block was significant for low-worriers, *F*_(1, 27)_ = 18.10, *p* < 0.001, partial η^2^ = 0.40, but there was no linear trend for block for high-worriers, *F*_(1, 27)_ = 2.49, *p* > 0.10.

Finally, the proportions of increasing option selections made by participants during the final 50 trials of the experiment were examined within each group to determine if participants learned to prefer one option significantly more than would be expected by chance (50%). Participants in the low-worry group (*M* = 0.61, *SE* = 0.03) selected the increasing option significantly more often than expected by chance, *t*_(27)_ = 3.05, *p* < 0.01, indicating a learned preference for the increasing option. Participants in the high-worry group selected the increasing option less often than expected by chance, although the difference was only marginally significant, *t*_(27)_ = −1.95, *p* < 0.10. We performed the same analysis on the proportion of increasing option selections during the first 50 trials of the experiment. Both low-worriers, *t*_(27)_ = −3.31, *p* < 0.01, and high-worriers, *t*_(27)_ = −6.62, *p* < 0.001, selected the increasing option significantly less often than expected by chance, which suggests that both groups initially favored the decreasing option but only low worriers eventually learned to select the increasing option more often than the decreasing option.

#### Model-based analyses

We fit a HYBRID RL model similar to that recently used in other work which individuals make decisions based on the immediate version future effects of each outcome (Eppinger et al., 2013). The model estimates both *model-free* reward values which are based on the immediate rewards received after selecting each option and *model-based* reward values which are based on the future value of each action, including how each action affects one's future state. In addition we also fit a Baseline or null model that assumes a stochastic response process (Yechiam and Busemeyer, 2005; Worthy and Maddox, 2012; Worthy et al., 2012).

The HYBRID RL model assumes that participants observed the hidden state (*s*) on each trial, which was equivalent to the number of times the increasing option had been selected over the previous ten trials. The model values options based on both the probability of reaching a given state on the next trial (*s′*) by selecting action *a* (the model-based component), and on the rewards experienced in each state (the model-free component). This model is similar to other models that have assumed that subjects use state-based information to determine behavior (Gureckis and Love, 2009; Gläscher et al., 2010; Daw et al., 2011; Eppinger et al., 2013). Following each trial in state *s* and arriving in state *s′* after having taken action *a* the model computes a state prediction error (SPE):

(1) $$\delta_{SPE}=1-T(s,a,s^{\prime })$$

Next, the model updates the state transition probability:

(2) $$T\left(s,a,s^{\prime }\right)=T\left(s,a,s^{\prime }\right)+\eta \delta_{SPE}$$

Here η is a free parameter that controls the learning rate, or effect of recent outcomes, for the state-transition probabilities. The state-transition probabilities for all other states not arrived at (denoted as *s*″) are reduced according to:

(3) $$T\left(s,a,s^{\prime \prime }\right)=T\left(s,a,s^{\prime \prime }\right)\cdot (1-\eta )$$

This ensures that all transition probabilities at a given state sum to 1.

The model also tracks the model-free expected reward values for each action in each state (*Q_MF_*(*s,a*)) using a SARSA learner [State-Action-Reward-State-Action (Morris et al., 2006; Gläscher et al., 2010)]. On each trial the model computes the reward prediction error (δ_*RPE*_):

(4) $$\delta_{RPE}=r-Q_{MF}(s,a)$$

The prediction error is then used to update the expected value for the current state action pair:

(5) $$Q_{MF}(s,a)=Q_{MF}(s,a)+\alpha \delta_{RPE}$$

Here α is a free parameter that represents the learning rate, or effect of recent outcomes,^1^ for state-action pairs on each trial. The model also has the ability to allow reward information gained for actions in a specific state to be generalized across states which has been shown to improve model fits in the same task (Gureckis and Love, 2009). For each state other than the state on the current trial (denoted as s^*^) the *Q_MF_* value for the same action selected on the current trial is updated:

(6) $$Q_{MF}(s^{*},a)=Q_{MF}\left(s*,a\right)+\theta \left(5*r-Q_{MF}\left(s*,a\right)\right)$$

Here θ represents the degree to which the rewards received on each trial are generalized to the same action in different states.

After updating state-transition probabilities and expected reward value information the model the computes a *model-based* value for each action in each state (*Q_MB_*(*s,a*)) using a FORWARD learner that incorporates the state-transition probabilities and the Bellman equation to determine the future value of each action (Gläscher et al., 2010; Eppinger et al., 2013). In this task there are three possible states that participants will transition to on the next trial (*s′*) following action a on the current trial (they can stay in the same state or move up or down one state). We estimated the *Q_MB_* value for each state-action pair by the following equation:

(7) $${\text{Q}}_{MB}\left(s,a\right)=\sum_{s^{\prime }(s-1)}^{s^{\prime }(s+1)}T\left(s,a,s^{\prime }\right)*\max[{\text{Q}}_{MB}\left(s^{\prime },a^{\prime }\right)]$$

This function multiplies the probability of transitioning to each possible state on the next trial, having taken action *a* in trial *t*, by the maximum expected reward in state *s′* for either action.

The model then determines a net value for each action (*Q_Net_*(*s,a*)) by taking a weighted average of the model-based and model-free expected values:

(8) $$Q_{Net}(s,a)=\omega \cdot Q_{MB}\left(s,a\right)+\left(1-\omega \right)\cdot Q_{MF}\left(s,a\right)$$

Where ω is a free parameter that determines the degree to which choices are based on the model-based vs. model free components of the model.

Finally, the probability of selecting each action is determined using the Softmax rule:

(9) $$P(a,t)=\frac{e^{\left[\beta \cdot [Q_{Net}(s,a)+\pi \cdot rep(a)\right]}}{\sum_{j=1}^{n}e^{\left[\beta \cdot [Q_{Net}(s,j)+\pi \cdot rep(j)\right]}}$$

Here β is an inverse temperature parameter that determines the degree to which participants exploit the option with the highest expected value. Larger β estimates are indicative of more consistently selecting the highest valued option, and as β approaches 0 each option is selected randomly. The autocorrelation, or perseveration parameter, π, accounts for tendencies to perseverate (π > 0) or switch (π < 0) regardless of the outcome on the last trial. For the option that was selected on the prior trial *rep(a)* is set to 1, and for all other options *rep(a)* = 0 (Lau and Glimcher, 2005; Daw et al., 2011; Eppinger et al., 2013). In total the RL model included six free parameters: η, α, θ, ω, β, and π.

The Baseline model had one free parameter for the two choice task which represents the probability of selecting option *a*. This parameter is subtracted from 1 to determine the probability of selecting the other option. For the four-choice task the Baseline model had three free parameters representing the probability of selecting three of the four options on any given trial. The probability of selecting the fourth option is 1 minus the sum of the probabilities of the three other options.

***Modeling results***. We fit each participant's data individually with the HYBRID RL, and Baseline models detailed above. The models were fit to the choice data from each trial by maximizing log-likelihood. We used Akaike's Information Criterion (AIC) compare the relative fit of the RL model to that of the Baseline model (Akaike, 1974). AIC is used to compare models with different numbers of free parameters. AIC penalizes models with more free parameters. For each model, *i*, AIC is defined as:

(10) $$AIC_{i}=-2logL_{i}+2V_{i}$$

where *L_i_* is the maximum likelihood for model *i*, and *V_i_* is the number of free parameters in the model. Smaller AIC values indicate a better fit to the data. To assess the degree to which participants were fit best by the RL model relative to the Baseline model we computed a Relative Fit metric by subtracting the AIC of the RL model from that of the Baseline model:

$$Relative\;Fit_{RL}=AIC_{Baseline}-AIC_{RL}$$

Larger values indicate a better fit of the model compared to the baseline model (Worthy et al., 2012).

The RL model provided a much better fit to the data than the Baseline model for both low and high-worry participants. However, *Relative Fit_RL_* values were significantly higher for low-worry (*M* = 98.87, *SE* = 12.29) than for high worry participants (*M* = 51.64, *SE* = 9.66), *t*_(54)_ = 3.02, *p* < 0.01. A multiple regression with worry, anxiety, positive mood, and negative mood entered as predictors of the relative fit of the RL model indicated that worry negatively predicted *Relative Fit_RL_* values, β = −0.36, *p* < 0.05, although the overall ANOVA for the regression model did not reach significance, *F*_(4, 51)_ = 1.93, *p* = 0.13. None of the other predictors reached significance.

Having established that the RL model provided a good account for the data compared to the Baseline model, we next examined the best fitting parameter values for low and high worry participants. These are listed in Table 2. We were particularly interested in the ω parameter that weighed the contribution of the model-based component of the model to participants' choices. Data from low-worry participants were best fit by significantly higher ω parameter values than data from high-worry participants, *t*_(54)_ = 3.78, *p* < 0.001. A multiple regression with worry, anxiety, positive mood, and negative mood entered as predictors of ω parameter values showed that worry was a significant predictor, β = −0.58, *p* < 0.001. The overall model was significant, *F*_(4, 51)_ = 4.85, *p* < 0.01, and none of the other predictors reach significance.

**Table 2:** **Average Best-Fitting Parameter Estimates for the RL Model**.

	**Low worry**	**High worry**
State learning rate (η)	0.54 (0.45)	0.54 (0.43)
Reward learning rate (α)	0.45 (0.44)	0.38 (0.47)
Reward generalization rate (*uptheta*)	0.13 (0.29)	0.19 (0.28)
Model-based weight (ω)	0.94 (0.08)^***^	0.71 (0.31)^***^
Inverse temperature (β)	1.91 (2.04)	1.32 (1.92)
Perseveration (π)	5.32 (6.89)	5.73 (8.24)

*Standard deviations are listed in parentheses*.

*** *Significant difference at p < 0.001 level*.

We did not find any differences between low and high worriers for any of the other parameters of the RL model.

Finally, we also performed a cross validation of the model by using the best-fitting parameter sets from half of the participants in the low worry and high worry groups to predict the performance for the other half of participants in each group. For both low and high worriers we performed 1000 simulations using the RL model by sampling, with replacement a set of best-fitting parameters from one subject for each simulation. This is similar to the parametric boot-strap cross fitting analyses we have performed in other work (Worthy et al., 2012, 2013).

**Figure 4:**
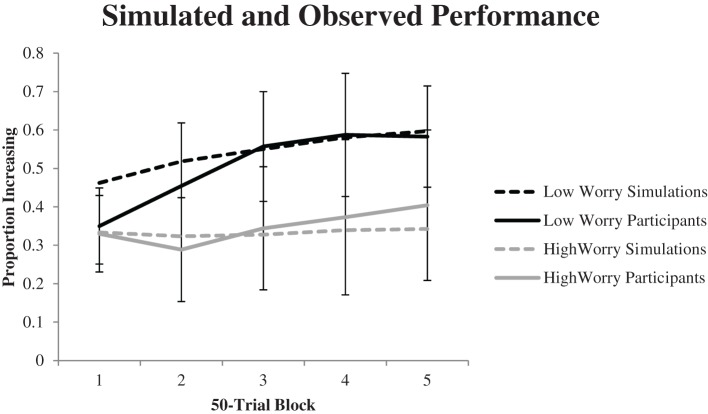
**Proportion of Increasing option selections from the cross validation analysis**. Error bars represent 95% confidence intervals. Best-fitting parameter values from fourteen participants within each of the high and low worry groups were used to perform the simulations, and the simulated results are shown compared to the actual performance for the other thirteen participants within each group.

Figure 4 shows the proportion of Increasing option selections from the simulations compared to the actual performance from the participants in each group whose best-fitting parameters were not used to perform the simulations. Overall the simulated performance aligned well with the performance from the observed participants. However, the model did over-predict Increasing option selections within the first 50 trial block of the task for low-worry participants, although that was the only block where the simulated results were outside of the appropriate 95% confidence interval from the observed participants.

### Discussion

The results of Experiment 1 suggest that high levels of worry were associated with a reduced prospection and an increased preference for the more immediately rewarding option during decision making. Interestingly we found that worry, anxiety, and negative mood scores were all positively correlated with one another, but worry was the only variable associated with decision-making behavior, and the association between worry and performance remained strong after partialing out the other factors. Low-worry participants learned to prefer the increasing option, which led to smaller immediate rewards but larger delayed rewards, more than high-worry participants, and more than expected by chance. Our modeling results suggest that low worry participants showed a greater tendency to select options based on how selecting those options would improve their future state. Parameter estimates from the model were also able to mimic the performance of low and high worry participants, as shown in our cross validation analysis. Data from low worry participants were best fit by higher ω parameter estimates which suggests that they exhibited a more model-based rather than a model free decision-making style. This led them to select the increasing option, which improved their future state more than the decreasing option that maximized their immediate reward.

While the effect of worry on decision-making behavior was quite robust, this was, nevertheless, just one experiment where we found these effects. In order to conceptually replicate and extend our findings we had participants perform a delay discounting task where they reported their preferences for receiving smaller amounts of money immediately over a larger amount of money after different delay periods.

## Experiment 2

In Experiment 2 we sought to directly examine how worry affected preferences for immediate and delayed rewards by using the DDQ where participants make hypothetical choices between $10 available after a specified delay or a smaller amount available immediately (Richards et al., 1999). One difference between the two experiments was that in Experiment 1 participants were required to make decisions from experience where information about the immediate and long-term rewards provided by each option had to be learned from sampling each option, while the DDQ used in Experiment 2 required decision making from description where descriptive information about each option was given beforehand and participants did not have to learn the consequences of selecting each option over the course of the task (Hertwig and Erev, 2009).

### Methods

#### Participants

Sixty-seven young adults enrolled at Texas A&M University completed the experiment for partial fulfillment of a course requirement.

#### Materials and procedure

Participants performed the Experiment on PCs using Psychtoolbox for Matlab (version 2.5). The experiment was approved by the Institutional Review Board. Participants were asked to complete the same questionnaires used in Experiment 1 before the experiment began (PSWQ, PANAS, and BAI).

Participants were told that they would be repeatedly asked whether they would prefer a smaller amount of money now or a larger amount of money ($10) after a specified delay (1, 2, 30, 180, or 365 days). They were told that these questions were hypothetical, but to try to answer as if they would actually be receiving the money. The task used an adjusting procedure where, for each delay period participants were initially offered $1 immediately vs. $10 after the specified delay, and the immediate reward that was offered was increased by $1 on the next trial until the immediate and delayed rewards were equal. This procedure allows for the derivation of an indifference point for each delay period which is the smallest amount of money an individual chose to receive immediately instead of the $10 offered after the delay. Lower indifference points indicate greater discounting of delayed rewards.

### Results

We used an area-under-the-curve (AUC) measure, as specified by Myerson et al. (2001) as our measure of participants' preference for delayed vs. immediate rewards. Smaller AUC values indicate greater discounting, or greater preference for immediate reward, while larger values indicate reduced discounting, or greater willingness to forego smaller immediate rewards in favor of larger delayed rewards.

**Figure 5:**
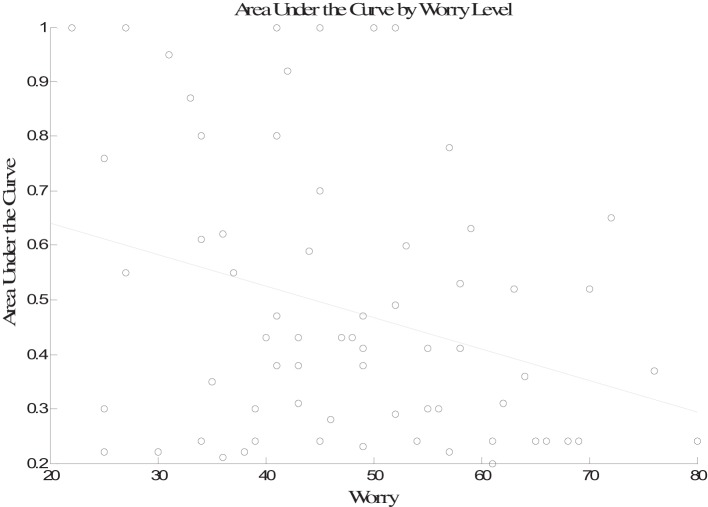
**Delay discounting score (area under the curve) plotted as a function of worry**.

The worry, anxiety, and positive and negative affect scores for each participant were entered as predictors in a linear regression with AUC values used as the outcome variable. Worry was significantly negatively associated with AUC, as were positive and negative mood. Table 3 lists the correlation coefficient matrix. Anxiety was also negatively associated with AUC, although the difference was not significant. Worry, anxiety, and negative mood were strongly correlated with one another as in Experiment 1. The results of the multiple regression indicate that worry (β = −0.29, *p* < 0.05) and positive mood (β = −0.28, *p* < 0.05)^2^ were significant predictors of participants' preferences for immediate vs. delayed rewards after accounting for other predictors, while anxiety (β = 0.05, *p* > 0.10) and negative mood (β = −0.16, *p* > 0.10) did not uniquely predict preferences for immediate vs. delayed rewards. Partial correlations for worry (*r* = −0.28, *p* < 0.05), and positive mood (*r* = −0.28, *p* < 0.05) were also significant. The association between worry and AUC is plotted in Figure 5.

**Table 3:** **Correlations Between Area Under the Curve (AUC) and Individual Differences in Experiment 2**.

		**1**	**2**	**3**	**4**	**5**
1	AUC	–	–	–	–	–
2	Worry	−0.31^**^	–	–	–	–
3	Anxiety	−0.20^**^	0.40^**^	–	–	–
4	Negative mood	−0.31^**^	0.36^**^	0.45^***^	–	–
5	Positive mood	−0.29^**^	−0.07	0.20	0.15	–

*Greater Area Under the Curve is indicative of reduced discounting or reduced preference for immediate over delayed rewards*.

** *p < 0.01*,

*** *p < 0.001*.

**Figure 6:**
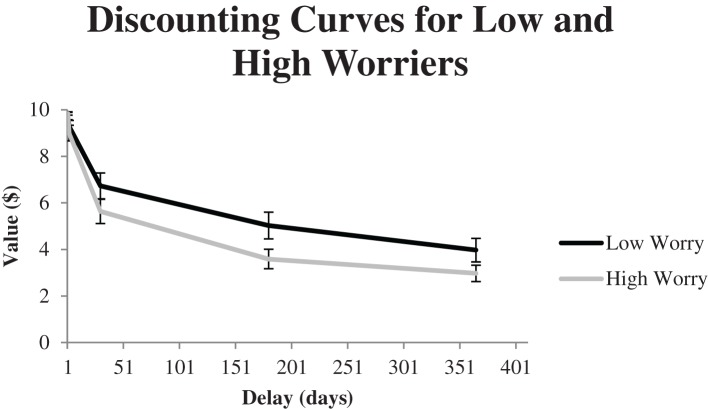
**Discounting curves for low and high worry participants for five different delays to the $10 standard value in Experiment 2**. These curves represent the mean indifference values for the standard as a function of delay.

To further examine the relationship between worry and delayed discounting, participants were split into high and low-worry groups based on a median split (Median = 47; Range = 22–80). There were 33 low-worry participants and 34 high-worry participants. Figure 6 shows the discounting curves for low and high worry participants. An independent samples *t*-test showed a significant effect of worry on AUC scores, *t*_(65)_ = 2.09, *p* < 0.05. Low-worry participants (*M* = 0.54, *SD* = 0.28) had significantly higher AUC scores than high-worry participants (*M* = 0.42, *SD* = 0.21), which suggests greater discounting of delayed rewards for high-worry participants.

## General discussion

The results across two different decision-making experiments suggest that high levels of worry reduce prospection and enhance preference for immediate over delayed reward. We argue that these results are due to worry leading to reduced preferences for rewards that may occur in the future, which are likely viewed as less certain. Experiment 1 required individuals to learn the immediate and delayed consequences of each choice from experience whereas Experiment 2 explicitly queried participants regarding whether they would prefer a smaller immediate reward or a larger delayed reward. Relative to low worriers, high worriers demonstrated enhanced preference for immediate reward in both experiments. Our results also suggest that worry, rather than anxiety, or negative mood, was the variable most strongly associated with decision-making behavior despite strong associations between worry, anxiety, and negative mood in both experiments. Thus, we can conclude that worry, rather than negative affect in general, has an independent effect on prospection in decision-making.

Our results do not support the view that high worriers' enhanced future orientated thinking styles increase prospection and lead them to prefer maximizing delayed reward over immediate reward (Borkovec et al., 1983; Brown et al., 1993). However, one thing to note is that our experiments required participants to maximize rewards that were gains, rather than to minimize losses or negative outcomes. One possibility is that the enhanced future-oriented thinking styles of non-worriers are specific to the consideration of negative future events. While our results suggest that high worriers favor maximizing immediate rather than delayed rewards when rewards are positively valued, high worriers may favor outcomes that minimize future losses more than outcomes that minimize immediate losses. One recent study found individuals with Generalized Anxiety Disorder were better able to avoid options with greater long-term losses in a modified version of the Iowa Gambling Task (IGT; Mueller et al., 2010). However, the IGT is distinct from the tasks we have employed in the present work in that it does not directly pit options that are more immediately rewarding against options that are more rewarding in the future. It is possible that more anxious individuals may react differently in situations where the context is more risk-oriented by including losses in the decision framework, while worriers may be more focused on minimizing uncertainty. Consequently, future work should examine how negative emotions like worry affect attention to both immediate and delayed losses. One possibility is that higher levels of worry are associated with greater attention to delayed losses. Alternatively, high levels of worry could simply enhance tendencies to avoid losses regardless of whether they occur immediately or at some point in the future.

One unexpected finding was that positive affect predicted greater discounting of delayed rewards in Experiment 2. While we did not predict this a priori, recent work has also found a link between positive affect and poor decision-making (including delay discounting). For example, Hirsh and colleagues found that positive affect was associated with enhanced discounting in extraverted individuals (Hirsh et al., 2010). In addition, very high positive affect has been shown to increase risk-taking behaviors, including drug use, sexual encounters and gambling (Cyders and Smith, 2008) and it is posited that increased positive affect can interfere with orientation to toward pursuit of long-term goals (Dreisbach and Goschke, 2004) which may result in greater orientation for the present and less focus on future goals/outcomes. While our finding of a positive association between positive mood and discounting is exploratory, and we did not measure any personality variables, future work should consider whether the effect of positive mood on discounting is robust, and whether it interacts with personality variables.

While our results are consistent with the hypothesis that worry is associated with reduced prospection during decision-making, it's important to note that our results could be due to some other mechanism unrelated to prospection. However, our results clearly demonstrate that worry is associated with enhanced preference for immediate vs. delayed rewards. The tasks we used both centered on decision-making situations where immediate and future rewards are placed at odds, and high levels of worry were associated with greater discounting of delayed reward in both situations. Future work should aim to further our understanding of how worry and other emotions affect thinking about future states and actions, and how emotion affects behavior and cognition, more broadly.

Worry is a commonly experienced emotion and many people suffer from excessive worry despite realizing that it is not always productive (Freeston et al., 1994). Despite worry being a future-oriented construct and related to a desire to avoid negative future events, our results suggest that high levels of worry may actually impair people's ability to engage in prospection and make the best long-term decisions. High worriers tended to maximize immediate reward at the expense of larger rewards in the future. Future work should further address the relationship between worry and prospection, including how the bias toward immediate reward might be attenuated in high worry-individuals when it proves counter-productive.

### Conflict of interest statement

The authors declare that the research was conducted in the absence of any commercial or financial relationships that could be construed as a potential conflict of interest.

## Footnotes

^1^One important point to note is that these learning rate parameters do not necessarily indicate better learning in the sense that participants learned the task better, but are more indicative of the effect of recent outcomes (Hochman et al., 2010). Higher estimates of these parameters would suggest that participants utilized recent outcomes to a greater extent when determining the expected value of each action.

^2^The negative association between positive mood and AUC was an unpredicted result that we will discuss in greater detail in the General Discussion.
